# Supplementary material for: Sustainable Esterification of a Soda Lignin with Phloretic Acid
Source: Polymers (Basel). 2021 Feb 21;13(4):637. doi: 10.3390/polym13040637 (PMC7924587; doi:10.3390/polym13040637)
Supplement: Supplementary file 1 [file polymers-13-00637-s001.pdf]

polymers

MDPI

Electronic supporting information

Sustainable Esterification of a Soda Lignin with Phloretic Acid

Antoine Adjaoud [1,2], Reiner Dieden [1], Pierre Verge [1,*]

[1] Luxembourg Institute of Science and Technology, Materials Research and Technology Department, 5 Avenue des Hauts-Fourneaux, L-4362 Esch-sur-Alzette, Luxembourg; antoine.adjaoud@list.lu (A.A.); reiner.dieden@list.lu (R.D.)
[2] University of Luxembourg, 2, Avenue de l'Université, L-4365 Esch-sur-Alzette, Luxembourg
* Correspondence: pierre.verge@list.lu (P.V.)

Keywords: lignin; sustainable; esterification; solubility

Table of figures

Table S1 Thermogravimetric analysis of the various grades of Protobind® lignin ……………….........................………...2

Table S2 Assignments of the lignin ^{13}C–^{1}H correlation peaks in the 2D HSQC spectra of P2400 and P2400-PA……….......3

Table S3 Assignments of the lignin ^{13}C–^{1}H correlation peaks in the 2D HMBC spectra of P2400-PA....................................4

Table S4 Effect and p-values of the individual variables and their first order interaction effects for the esterification of P2400 with PA...6

Table S5 Hansen partial solubility parameters of the solvent used for the solubility assays …..................................……..7

Figure S1 TGA and DTG curves of Protobind® lignin……..........................……………………..………………………...2

Figure S2 Aromatic region in the 2D HMBC NMR spectra of and P2400-PA (δ_C/δ_H: 125–180/2.4–4.4)..................................4

Figure S3 ^{31}P NMR spectrum of P2400 (R= -OMe, -O-lignin, lignin)………...……………………….........................…..4

Figure S4 FTIR spectra of P2400 and P2400-PA ……………………………………….............................……………...5

Figure S5 Linear predictive model ……………………………………………………..............................…...……...5

Figure S6 Surface response associated to a) c/ n, b) t/n and c) c/t critical variables………...............................……………...6

Figure S7 Weight average molecular weight of P2400 and P400-PA………………...........................…………………......8

Figure S8 DSC thermogram of P2400 and P400-PA…………………………..….……….............................……………...8

➢ Thermogravimetric analysis of Protobind® grades

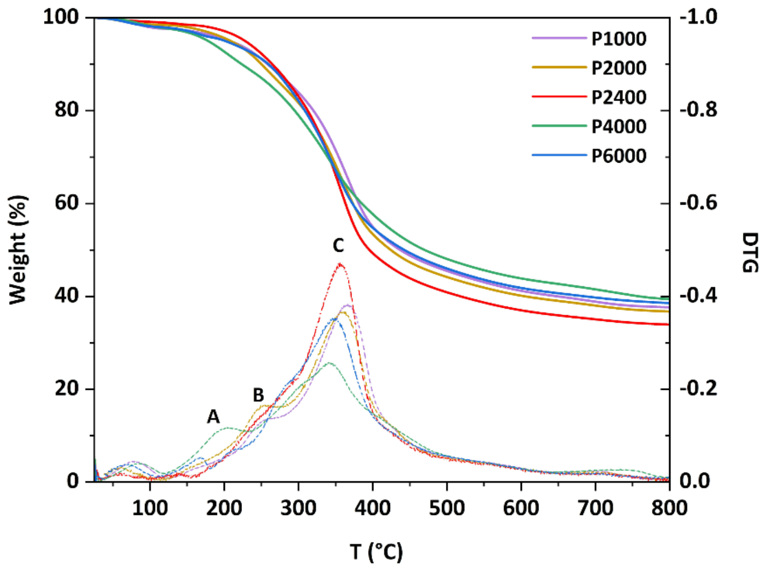

Figure S1 TGA and DTG curves of Protobind® lignin

TGA curves of the different grades of the Protobind® lignin are reported in Figure S1. The moisture content, the onset of thermal degradation (T_{onset}), the major degradation temperatures (A, B, C), and the char residue at 800 °C are gathered in Table S1. The DTG curves of the different grades of Protobind® lignin exhibited roughly similar decomposition pattern that can be sundered in three degradation ranges: A around 180°C, B around 300°C and the major degradation D around 350°C. The onset of the degradation temperature depends on the Protobind® grade. Although the highest T_{onset} is observed for the grade P2400 (157 °C), this grade exhibits the lowest char yield (33.9 %). The other grades of Protobind® lignin displayed a char yield around 37-38 %, with the highest obtained for the grade P4000 (39.4 %).

Table S1 Thermogravimetric analysis of the various grades of Protobind® lignin (10 °C.min^{-1}, N_2)

Protobind® grades	[a] T_{onset} (°C)	[a] Major degradation temperature (°C)			[b] Char (%)
		A	B	C	
P1000	130	-	259	369	37.6
P2000	124	-	247	361	36.7
P2400	157	-	299	359	33.9
P4000	121	207	302	342	39.4
P6000	115	169	283	348	38.5

[a] determined from the first derivative, [b] at 800 °C.

➢ Structural characterization of the esterified lignin

Table S2 Assignments of the lignin ^{13}C–^{1}H correlation peaks in the 2D HSQC spectra of P2400 and P2400-PA

Label	δ_C/δ_H (ppm)	Assignments	P2400	P2400-PA
B_3	30.0/2.75	C_3–H_3 in substructure **B**		x
B_2	36.1/2.57	C_2–H_2 in substructure **B**		x
C_β	54.2/3.09	C_β–H_β in resinol β- β′ substructure **C**	x	x
$–OCH_3$	56.5/3.76	C–H in methoxyl **–OMe**	x	x
A_x	60.8/3.52	C_x–H_x in substructure **A**	x	
$B_{x'}$	63.8/4.12	$C_{x'}$–$H_{x'}$ in substructure **B**		x
$A_{\text{-O-Alk}}$	70.3/3.52	$C_{\text{-O-Alk}}$–$H_{\text{-O-Alk}}$ in substructure **A**	x	
$B_{\text{-O-Alk}}$	70.3/3.52	$C_{\text{-O-Alk}}$–$H_{\text{-O-Alk}}$ in substructure **B**		x
C_γ	71.6/4.20;3.82	C_γ–H_γ in resinol β- β′ substructure **C**	x	x
X_2	73.1/3.13	C_2–H_2 in xylan substructure **X**	x	x
X_3	74.6/3.33	C_3–H_3 in xylan substructure **X**	x	x
X_4	75.9/3.58	C_4–H_4 in xylan substructure **X**	x	x
$S_{2,6}$	104.2/6.71	$C_{2,6}$–$H_{2,6}$ in syringyl units **S**	x	x
$S'_{2,6}$	106.8/7.27	$C_{2,6}$–$H_{2,6}$ in α-oxidized syringyl units **S′**	x	
Fa_2	111.2/7.41	C_2–H_2 in ferulate **Fa**	x	
G_2	112.6/6.83	C_2–H_2 in guaiacyl units **G**	x	x
B_6	115.6/6.69	C_6–H_6 in substructure **B**		x
G_5	115.6/6.70	C_5–H_5 in guaiacyl units **G**	x	x
Pc_β	115.8/6.32	C_β–H_β in *p*-coumarate **Pc**	x	
Fa_β	116.1/6.40	C_β–H_β in ferulate **Fa**	x	
G_6	119.3/6.78	C_6–H_6 in guaiacyl units **G**	x	x
$H_{2,6}$	128.8/7.06	$C_{2,6}$–$H_{2,6}$ in *p*-hydroxyphenyl units **H**	x	x
B_5	129.7/7.02	C_5–H_5 in substructure **B**		x
$Pc_{2,6}$	130.6/7.53	$C_{2,6}$–$H_{2,6}$ in *p*-coumarate **Pc**	x	
Pc_α	144.7/7.55	C_α–H_α in *p*-coumarate **Pc**	x	

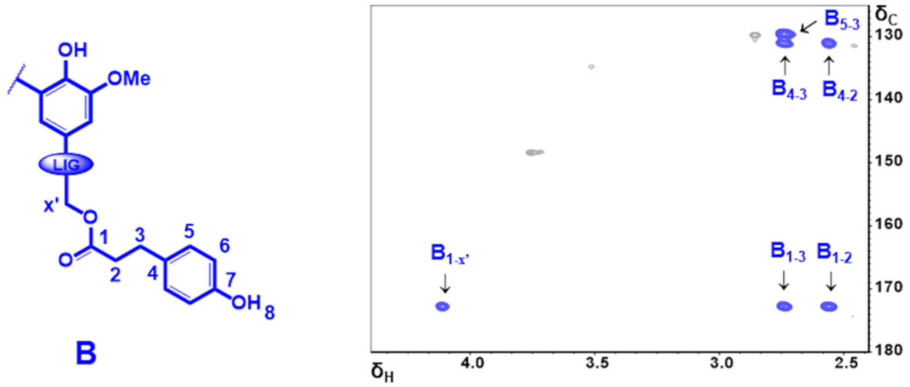

Figure S2 Aromatic region in the 2D HMBC NMR spectra of P2400-PA (δ_C/δ_H: 125–180/2.4–4.4). Signal assignments are reported in Table S3.

Table S3 Assignments of the lignin ^{13}C–^{1}H correlation peaks in the 2D HMBC spectra of P2400-PA

Label	δ_C/δ_H (ppm)	Assignments
B_{5-3}	129.7/2.75	C_5–H_3 in substructure **B**
B_{4-2}	131.0/2.57	C_4–H_2 in substructure **B**
B_{4-3}	131.0/2.75	C_4–H_3 in substructure **B**
B_{1-2}	172.8/2.57	C_1–H_2 in substructure **B**
B_{1-3}	172.8/2.75	C_1–H_3 in substructure **B**
$B_{1-x'}$	172.8/4.12	C_1–$H_{x'}$ in substructure **B**

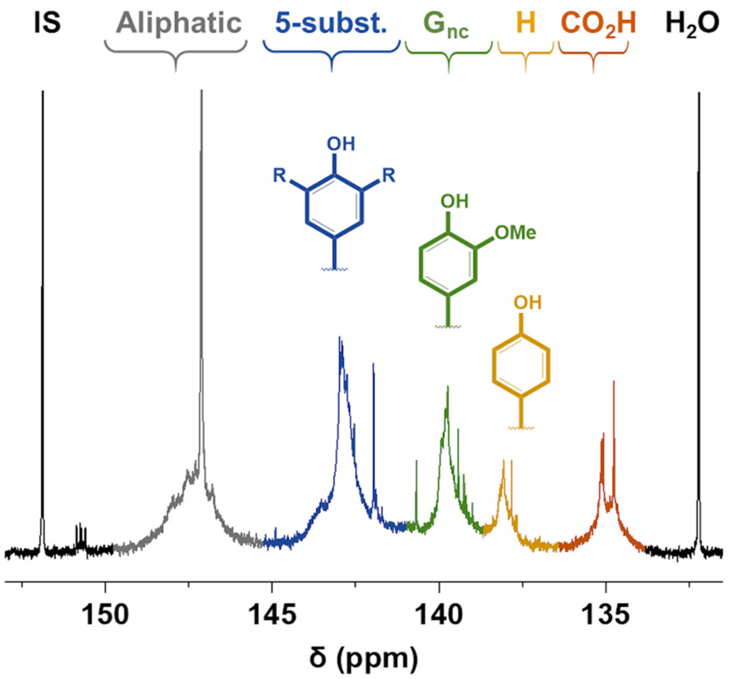

Figure S3 ^{31}P NMR spectra of P2400 (R= -OMe, -O-lignin, lignin)

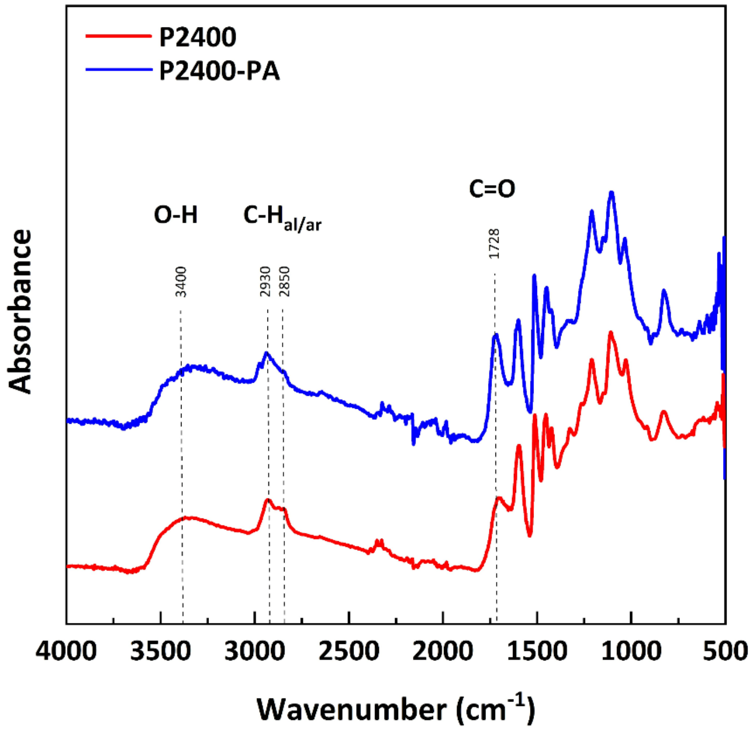

Figure S4 FTIR spectra of P2400 and P2400-PA

➢ <u>**Design of Experiment methodology**</u>

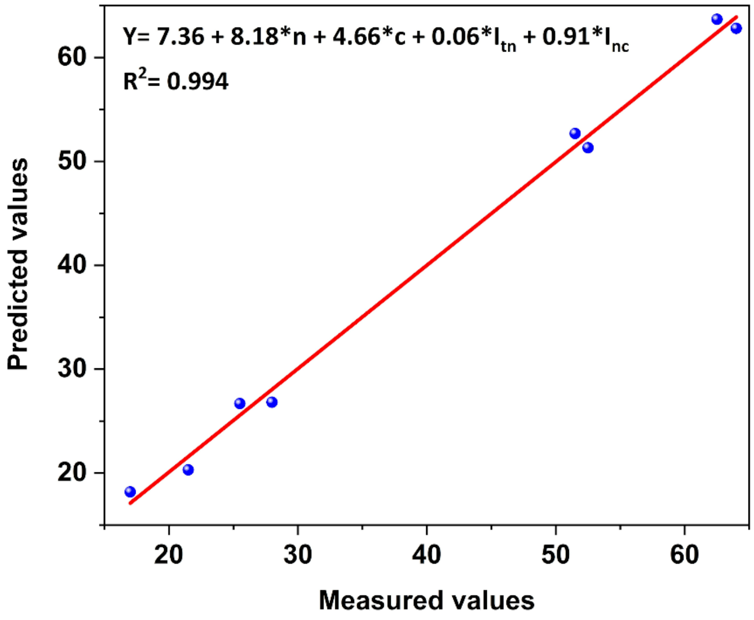

Figure S5 Linear predictive model, equation **(4)**

<u>Evaluation of the predictive model (EL-9)</u>: Y= 7.36 + 8.18*3 + 4.66*1.5 + 0.06*30*3 + 0.91*3*1.5 = 48.5 %

Table S4 Effect and p-values of the individual variables and their first order interaction effects for the esterification of P2400 with PA

Model	Factor	t	n	c	I_{tn}	I_{tc}	I_{nc}
Linear predictive model **(4)**	Effect	Ø	10.1019	3.2330	4.9669	Ø	-2.2669
	p	Ø	0.002	0.048	0.016	Ø	0.108

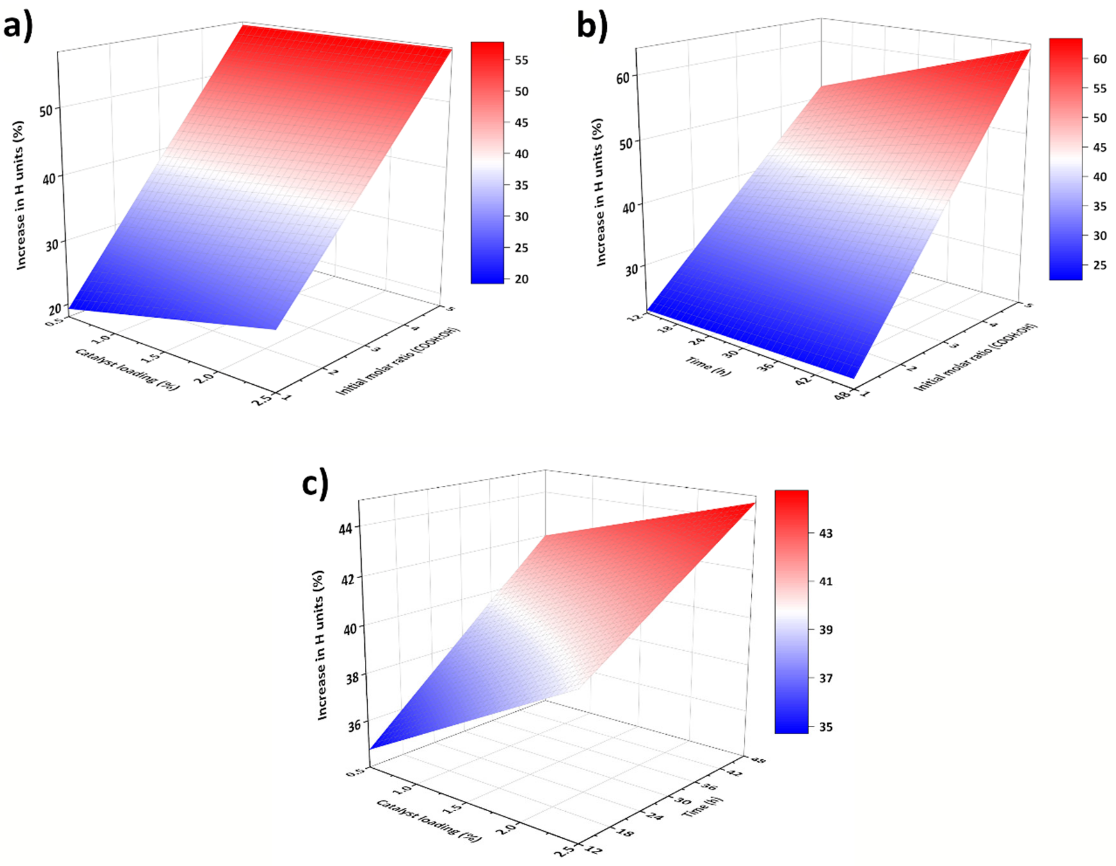

Figure S6 Surface response associated to a) c/n, b) t/ n and c) c/t critical variables

➢ **Physicochemical properties of the esterified lignin**

Table S5 Hansen partial solubility parameters of the solvent used for the solubility assays

Solvent	δ_D (MPa$^{1/2}$)	δ_P (MPa$^{1/2}$)	δ_H (MPa$^{1/2}$)
Acetone (ACE)	15.5	10.4	7.0
Acetonitrile (ACN)	15.3	18.0	6.1
Chloroform ($CHCl_3$)	17.8	3.1	5.7
Dichloromethane (DCM)	17.0	7.3	7.1
Diethyl ether (DET)	14.5	2.9	4.6
Dimethyl formamide (DMF)	17.4	13.7	11.3
Dimethyl sulfoxide (DMSO)	18.4	16.4	10.2
Dioxane (DIOX)	17.5	1.8	9.0
Ethanol (EtOH)	15.8	8.8	19.4
Ethyl acetate (ETAC)	15.8	5.3	7.2
Methanol (MeOH)	14.7	12.3	22.3
Methyl ketone (MEK)	16.0	9.0	5.1
Pyridine (PYR)	19.0	8.8	5.9
Tetrahydrofuran (THF)	16.8	5.7	8.0
Toluene (TOL)	18.0	1.4	2.0
Water (H_2O)	15.5	16	42.3

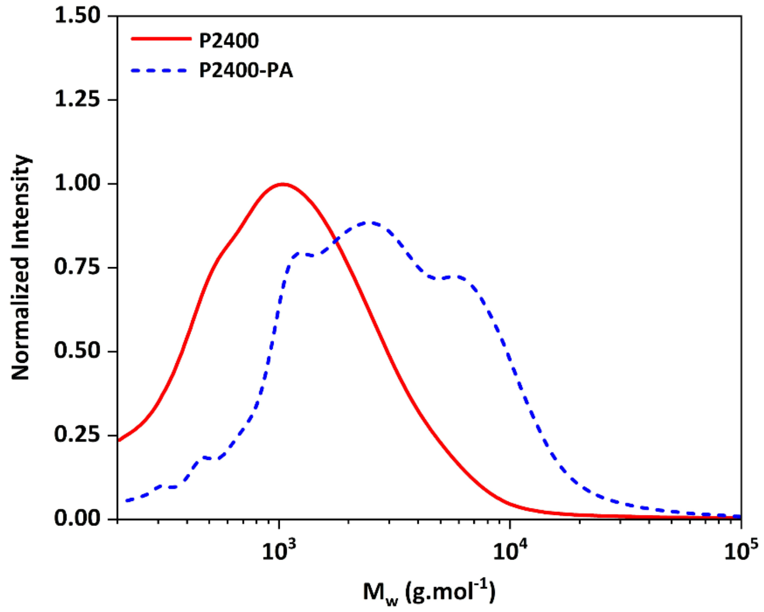

Figure S7 Weight average molecular weight of P2400 and P400-PA (4 mg.ml^{-1} in THF)

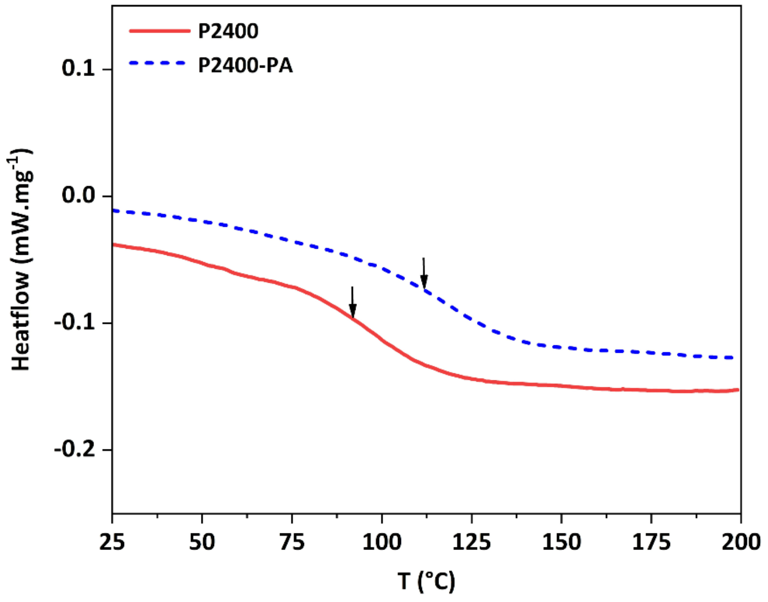

Figure S8 DSC thermogram of P2400 and P400-PA (10 °C.min^{-1}, N_2)
